# r2VIM: A new variable selection method for random forests in genome-wide association studies

**DOI:** 10.1186/s13040-016-0087-3

**Published:** 2016-02-01

**Authors:** Silke Szymczak, Emily Holzinger, Abhijit Dasgupta, James D. Malley, Anne M. Molloy, James L. Mills, Lawrence C. Brody, Dwight Stambolian, Joan E. Bailey-Wilson

**Affiliations:** Statistical Genetics Section, Inherited Disease Research Branch, National Human Genome Research Institute, National Institutes of Health, 333 Cassell Dr, 21224 Baltimore, USA; Clinical Trials and Outcomes Branch, National Institute of Arthritis and Musculoskeletal and Skin Diseases, National Institutes of Health, 1 AMS Circle, 20892 Bethesda, USA; Division of Computational Bioscience, Center for Information Technology, National Institutes of Health, 12 South Dr, 20892 Bethesda, USA; Department of Clinical Medicine, School of Medicine, Trinity College Dublin, 152-160 Pearse Street, 2 Dublin, Ireland; Division of Intramural Population Health Research, Eunice Shriver National Institute of Child Health and Human Development, National Institutes of Health, 6100 Executive Blvd, 20892 Bethesda, USA; Molecular Pathogenesis Section, Medical Genomics and Metabolic Genetics Branch, National Human Genome Research Institute, National Institutes of Health, 50 South Dr, 20892 Bethesda, USA; Department of Ophthalmology, University of Pennsylvania, 422 Curie Blvd, 19104 Philadelphia, USA; Current address: Institute of Medical Informatics and Statistics, University of Kiel, Brunswiker Str. 10, 24105 Kiel, Germany

**Keywords:** Machine learning, Random forest, Variable selection, Variable importance, Genome-wide association study, Genetic, SNP

## Abstract

**Background:**

Machine learning methods and in particular random forests (RFs) are a promising alternative to standard single SNP analyses in genome-wide association studies (GWAS). RFs provide variable importance measures (VIMs) to rank SNPs according to their predictive power. However, in contrast to the established genome-wide significance threshold, no clear criteria exist to determine how many SNPs should be selected for downstream analyses.

**Results:**

We propose a new variable selection approach, recurrent relative variable importance measure (r2VIM). Importance values are calculated relative to an observed minimal importance score for several runs of RF and only SNPs with large relative VIMs in all of the runs are selected as important. Evaluations on simulated GWAS data show that the new method controls the number of false-positives under the null hypothesis. Under a simple alternative hypothesis with several independent main effects it is only slightly less powerful than logistic regression. In an experimental GWAS data set, the same strong signal is identified while the approach selects none of the SNPs in an underpowered GWAS.

**Conclusions:**

The novel variable selection method r2VIM is a promising extension to standard RF for objectively selecting relevant SNPs in GWAS while controlling the number of false-positive results.

**Electronic supplementary material:**

The online version of this article (doi:10.1186/s13040-016-0087-3) contains supplementary material, which is available to authorized users.

## Background

In the last few years, more than one thousand single-nucleotide polymorphisms (SNPs) have been reproducibly associated with more than two hundred phenotypes and quantitative traits in genome-wide association studies (GWAS) [1]. These loci are usually identified by linear or logistic regression analysis which is performed separately for each SNP. The resulting *p*-values are then used to rank the SNPs and to select those with a *p*-value smaller than a pre-specified significance level which is adjusted for the large number of statistical tests. In such a scenario, comparable to analyses of other genomic data sets such as gene expression, *p*-values are not used in a confirmatory setting but rather as a screening tool to identify associated, i.e. important, SNPs while controlling the number of false positive findings.

Nonparametric, model-free statistical learning machines provide a promising alternative to classical, model-based statistical methods for the selection of important variables in high dimensional data sets. One major advantage is their ability to identify genetic variants that have a joint effect on the phenotype which makes more biological sense than the assumption of individual SNPs always acting independently. Indeed, the common variants that have been successfully identified in GWAS studies thus far appear to explain only a small proportion of the overall heritability [2].

Popular learning machines, such as random forests (RFs) [3], are known to be statistically optimal and are computationally efficient when run in parallel on distributed systems. RF is an ensemble method based on a large number of classification and regression trees trained on bootstrap samples and has been successfully applied to identify SNPs influencing susceptibility to disease [4–6].

RF provides variable importance measures (VIMs) that can be used to order and select the most predictive SNPs. But the actual importance values are difficult to interpret as they depend not only on the signal in the data but also on the parameters of the algorithm [7]. Usually, SNPs are ranked according to decreasing importance values and the top ranked SNPs are declared as important. The number of selected SNPs is often arbitrary and several approaches have been proposed to objectively determine a threshold. A classical statistical test could be used by estimating z-scores and calculating asymptotic *p*-values [8]. However, the power of this test depends on the number of trees, which is a tuning parameter in RF. Therefore, this method is not recommended [9].

As an alternative, the null distribution of the VIMs can be estimated by permuting phenotype status (see e.g. R package rfpermute). Unfortunately, this approach would require at least 1000 runs of RF and is therefore computationally prohibitive for GWAS data sets. Therefore, it is difficult to decide how many SNPs should be selected with the threshold being somewhat arbitrary. As a consequence, no clear criteria exist to decide if RF is able to identify any important SNP or if the study is underpowered. Indeed, simulations have shown that when the effects of the causal SNPs on the trait are low and/or sample size is not extremely large, then most of the SNPs with strongest VIMs are not causally related to the trait [10, 11]. Here we present a novel variable selection procedure called recurrent relative variable importance measure (r2VIM). Several runs of RF are performed each resulting in importance values calculated relative to the observed minimal importance score. Only SNPs with large relative VIMs in all of the runs are declared as important. GWAS data with realistic local linkage disequilibrium patterns were simulated to evaluate false-positives and empirical power compared to logistic regression. Analysis of two experimental GWAS, one that has a strong signal and another one that is underpowered, illustrate the applicability of our new method.

## Methods

### Random forest

RF is a machine learning approach that combines many classification and regression trees into a committee or ensemble [3]. Each tree is built using a bootstrap sample of the data set and at each node the optimal variable is selected from a random subset of all predictor variables. Majority voting over all trees is used to classify a sample using the ensemble. In addition to prediction, RF estimates VIMs that can be used to assess the predictive power of each variable. The most reliable measurement is the (unscaled) permutation importance [12] that measures the difference in prediction accuracy before and after permuting values of the variable, averaged over all trees.

### New variable selection method r2VIM

Our proposed variable selection method r2VIM is based on the permutation importance scheme, a standard component of RF. Our method has three components. First, instead of performing a single run of RF and selecting a few top ranked variables, we propose running RF several times with different random number seeds. Then, trees in each run over several forests will be slightly different leading to random variability in VIMs, where the randomness is partly sample based and partly seed based. Variables more predictive of the outcome will have relatively high importance scores in each of the runs, while other, less predictive variables will have only randomly high importance scores. The second component of the scheme is that variables with little predictive capacity will have importance values close to zero. It is useful to note that the variable importance values in RF will generate importances that may be negative. Therefore, since most SNPs in a GWAS setting are not expected to be associated with the disease, the smallest, usually negative, observed importance score across the variables, SNPs in a GWAS, can be used as an approximate estimate of the variability for variables with no predictive power. The related idea here is that noise variables will have importances randomly and symmetrically above and below zero. For each variable, we define a relative importance score by dividing its value by the observed absolute value of the minimal importance score. Hence all SNPs with a relative importance score larger than 1 (or in general a factor ***f***) could then be defined as important (see e.g. [13]), or more accurately, not unimportant. The last part combines the other two components by declaring only those SNPs as important that have relative importance scores  >  ***f*** in each of the runs.

The approach is implemented as a R package called r2VIM [14].

For all analyses presented in this paper we used ten runs and factors ***f*** = 1, 3, and 5. As shown in the results, using ***f***  =  1 identifies too many false-positive SNPs under the null hypothesis. That is, the simple observed minimum, negative importance value is not a good estimate of the variance of importances across noise features, while simple multiples of the observed minimum seem to do quite well.

### Simulation study 1

To evaluate our new variable selection method we first simulated genome-wide SNP data sets with realistic local linkage disequilibrium patterns and relatively large effects that depend on the MAF of the corresponding SNPs. Haplotypes from 381 European individuals provided by the 1000 genomes project [15] were used as input data for the software GWAsimulator [16] to simulate new haplotypes for a case–control study. 554,813 SNPs from the Illumina Human660W chip were selected and 10 replicates generated. We used total sample sizes of 2000 and 6000 with a balanced number of cases and controls.

To estimate the number of false-positive SNPs, a null hypothesis was simulated where case–control status was not dependent on the genotypes at any SNP but was assigned randomly. To demonstrate the feasibility of the new variable selection method, a simple alternative hypothesis based on a small number of common and independent SNPs with relatively large effects was generated. Case–control status was determined by nine independent causal SNPs, each with multiplicative (on relative risk level) main effects. For reasonable power, most of the causal SNPs were common with minor allele frequencies (MAF) of 0.3 or nearly 0.5 and relative risks for one minor allele was set to 1.5 or 1.3. In addition, three less common SNPs with MAF of 0.06 and a relative risk of 2 were included into the model. Detailed information about all nine SNPs can be found in Table 1. Effect sizes of SNPs are given as relative risks since it is not possible to specify odds ratios in the used simulation software. However, only odds ratios can be estimated in case–control studies which are equivalent to relative risks for rare diseases.

**Table 1:** Information about the nine causal SNPs under the alternative hypothesis in simulation study 1

SNP	MAF	RR	no SNPs strong LD	no SNPs moderate LD
11-103959987	0.474	1.3	0	1
22-28469630	0.488	1.3	4	8
17-9807099	0.496	1.3	0	0
1-240799543	0.312	1.5	0	0
7-45984820	0.312	1.5	0	2
5-130104076	0.323	1.5	12	18
14-67463012	0.062	2	2	31
18-34645639	0.062	2	0	1
3-2770509	0.064	2	0	1

Table shows SNP identifier in chromosome and position notation, minor allele frequency (MAF), relative risks (RR) and number of SNPs within a 1 Mb region that are in strong (*r* ^*2*^ > 0.8) or moderate LD (0.3 < *r* ^*2*^ ≤ 0.8)

### Simulation study 2

We performed a second simulation study focussing on the empirical power for a set of SNPs with the same effect on the phenotype but different MAFs and local LD patterns. We used the software SeqSIMLA2 [17] to simulate a region on chromosome containing 107,454 SNPs with MAF  >  0.05 and realistic LD patterns based on 10,000 haplotypes provided by the software that were generated using European samples of the 1000 genomes project [15]. Nine causal SNPs were selected based on regions of high, low or no LD as well as MAFs close to 0.1, 0.3 and 0.5. We simulated two scenarios, each with ten replicates. In the first one, each of the SNPs was assigned an OR of 1.3 and 3000 cases and 3000 controls were simulated. The second setting had more realistic effect sizes of 1.1, but 10,000 samples were generated for cases as well as controls.

### Experimental data

We selected two GWAS studies to illustrate application of the new method on real data sets. To compare results from a GWAS with a strong signal we used GWAS phenotype and SNP data from the Trinity Student Study (TRINITY) which examines traits related to folate and vitamin B12 metabolism in healthy young Irish individuals who were university students at Trinity College in Dublin (TCD), Ireland between 2003 and 2004. Eligible subjects were between 18 and 28 years of age at the time of study enrollment, did not report a serious medical condition, and were ethnically Irish. All study participants provided written informed consent. The Research Ethics Committee of the Dublin Federated Hospitals, which is affiliated with TCD, and the Institutional Review Board of the National Human Genome Research Institute gave ethical approval for the study. Further details of this study have been published previously [18–20]. The analyzed phenotype is total serum bilirubin (TBIL) measured as a quantitative trait. For illustration purposes, we selected individuals at the extremes of the distribution. 193 individuals with TBIL  > 17 and 241 individuals with TBIL  <  5 were defined as cases and controls, respectively. Since missing values pose a problem for RF, quality controlled SNPs were imputed with PLINK using CEU individuals from phase 2 of the HapMap project as the reference panel resulting in 873,565 common SNPs with complete genotypes.

As a negative control data set we chose a GWAS study with a relatively small sample size so that the power to identify a real effect is very low. Data are from the Age-Related Eye Disease Study (AREDS), that was initially designed as a long-term, multicenter, prospective study to assess the clinical course of age-related macular degeneration (AMD) and age-related cataract [21]. In addition to collecting natural history data, AREDS included a randomized clinical trial of high-dose vitamin and mineral supplements for AMD and a clinical trial of high-dose vitamin supplements for cataract [21–23]. Prior to study initiation, the protocol was approved by an independent data and safety monitoring committee and by the institutional review board for each clinical center. Written informed consent was obtained from all participants in accordance with the Declaration of Helsinki. AREDS participants were 55 to 80 years of age at enrollment and had to be free of any illness or condition that would make long-term follow-up or compliance with study medications unlikely or difficult. For the current analysis, a subset of the control group from the original AREDS study was included: 2000 Caucasian participants aged 60 and older who did not have AMD and were further screened to also exclude individuals with cataracts, retinitis pigmentosa, color blindness, other congenital eye problems, LASIK, artificial lenses, and other eye surgery. Mean spherical equivalent (MSE) of both eyes was calculated on study participants without either AMD or cataracts at the first study visit. A binary phenotype, hyperopia, defined 858 cases as those with MSE  ≥   + 1D and 602 controls with MSE  <  0D. Quality-controlled SNPs were imputed using MACH [24] based on HapMap phase 2 reference panel. To reduce the number of SNPs for analysis, LD pruning was performed using PLINK with pairwise *r*^*2*^ of 0.99 as threshold. 908,293 common SNPs with complete genotypes remained for analysis. Further details about the genotype data have been published previously [25, 26].

### Analyses

RF analyses were performed with RandomJungle [6] versions 1.2.365 and 2.0.0. Ten RF runs were performed for each data set or replicate using 1000 classification trees and about 20, 25 or 50 % of SNPs randomly selected at each node. The number of samples in terminal nodes was restricted to 5 or 10 % of the total sample size. To make the analyses computationally feasible, depth of trees was limited to 3. Important SNPs were selected using r2VIM with factors of 1, 3 and 5, i.e. only SNPs with relative importance scores  >  1, 3 and 5 in each of the ten runs were selected.

For comparison, standard statistical analysis of GWAS data was performed. A logistic regression model was fitted in PLINK versions v1.07 [27] and v1.90b3y [28] for each SNP separately. Similar to the variable selection method, SNPs with a *p*-value smaller than a pre-specified threshold were selected. For simulation study 1 as well as the experimental data sets, the genome-wide significance level of 5*10^−8^ was used, whereas for simulation study 2, a stringent Bonferroni based threshold of 0.05/100,000 = 5*10^−7^ was applied.

Type I errors and empirical power were estimated for each SNP separately using the proportion of replicates in which a particular SNP was identified by r2VIM or logistic regression. A SNP was declared a false-positive if it was not in LD with any causal SNP. We employed the clumping approach as implemented in PLINK to identify causal SNPs in LD with selected SNPs.

## Results

### Simulation study 1

**Fig. 1:**
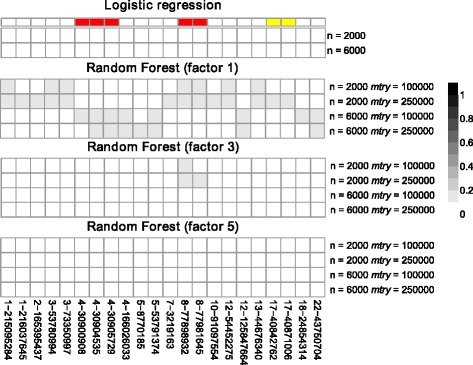
Heatmaps of type I error of single SNPs in simulation study 1. Shown are results for logistic regression and r2VIM with several factors in the different scenarios (different sample sizes and *mtry* parameters in RF). Columns correspond to SNPs that are selected in at least one approach and are ordered by chromosomal position. Type I error is color-coded in gray with white and black denoting 0 and 1, respectively. In addition, LD information is shown at the top with SNPs in high (*r* ^*2*^ > 0.8) and moderate LD (0.3 < *r* ^*2*^ ≤ 0.8) colored in red and yellow

Results under the null hypothesis with case–control status assigned randomly are shown in Fig. 1. As expected, no SNP reaches genome-wide significance in all ten replicates for logistic regression. In contrast, the number of false-positive SNPs identified by r2VIM depends on the factor that is used to define the threshold for declaring SNPs as important (see Fig. 1). If a liberal factor of 1 is used, between seven and 13 SNPs are selected across settings. Three and two SNPs on chromosomes 4 and 8 are highly correlated (pairwise *r*^*2*^  >  0.8), resulting in five to 12 independent regions. However, all SNPs have been selected in only one replicate and each SNP is selected either with a sample size of 2000 or 6000. In general, a smaller number of false-positive SNPs is identified for the larger sample size. If the factor is increased to 3, only the region on chromosome 8 is selected for the smaller sample size whereas none of the SNPs is found for the larger one. In addition, if the most stringent factor of 5 is used, type I error is well controlled since r2VIM declares none of the 500,000 SNPs as important.

**Fig. 2:**
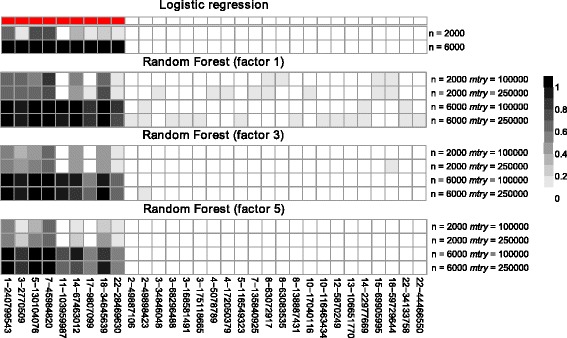
Heatmaps of empirical power of single SNPs in simulation study 1. Shown are results for logistic regression and r2VIM with several factors in the different scenarios (different sample sizes and *mtry* parameters in RF). Only the nine causal SNPs (marked in red on top) and false-positive SNPs that are uncorrelated to each causal SNP are shown in columns and ordered by chromosomal position. Empirical power is color-coded in gray with white and black denoting 0 and 1, respectively

Empirical power under the alternative hypothesis is summarized in Fig. 2 and Table 2. Detailed information about each SNP that was detected in at least one replicate and with at least one method is given in Additional file 1: Table S1. With logistic regression eight out of the nine causal SNPs have empirical power  >  0 for the smaller sample size. However, only the three common SNPs with relative risks of 1.5 have significant *p*-values in more than 5 replicates. In the larger data set, all causal SNPs are identified in all ten replicates. All other SNPs with significant *p*-values are in LD with one of the causal SNPs. r2VIM identifies seven and nine causal SNPs with a sample size of 2000 and 6000, respectively. However, power decreases from factor 1 to 3 and 5. The largest reduction in power is observed for the very common SNPs with small effects on chromosomes 17 and 22. Increasing the factor value also reduces the number of selected SNPs that are correlated with one of the causal SNPs. In concordance with results under the null hypothesis, using a factor of 1 results in identification of four to 13 false-positive SNPs (each observed in only a single replicate) that are not correlated with any of the causal SNPs and that are often located on other chromosomes. Interestingly, more false-positives are observed for the larger *mtry* value for both sample sizes. Again, the number is greatly reduced for a factor of 3 with one false-positive SNP identified with *mtry* = 250000. And only causal SNPs or SNPs correlated with causal SNPs are selected with a factor of 5.

**Table 2:** Number of SNPs in simulation study 1 with empirical power > 0

Method	*n*	*mtry*	Factor	Total	Causal	High LD	Mod LD	Low LD	FP
LR	2000			40	8	15	11	4	0
LR	6000			98	9	16	15	15	0
r2VIM	2000	100000	1	38	7	13	10	2	4
r2VIM	2000	100000	3	28	7	12	6	2	0
r2VIM	2000	100000	5	24	7	10	5	2	0
r2VIM	2000	250000	1	40	8	12	9	2	8
r2VIM	2000	250000	3	25	7	9	5	2	1
r2VIM	2000	250000	5	23	7	9	5	2	0
r2VIM	6000	100000	1	51	9	16	10	5	3
r2VIM	6000	100000	3	41	9	16	6	4	0
r2VIM	6000	100000	5	37	9	16	6	4	0
r2VIM	6000	250000	1	63	9	16	12	6	13
r2VIM	6000	250000	3	42	9	16	7	4	1
r2VIM	6000	250000	5	37	9	16	5	4	0

Shown are results for logistic regression (LR) and r2VIM. Columns denote method, sample size (*n*), mtry parameter and factor for r2VIM, total number of SNPs, number of SNPs in strong (*r* ^*2*^ > 0.8), moderate LD (0.5 < *r* ^*2*^ ≤ 0.8) and low LD (0.3 < *r* ^*2*^ ≤ 0.5) with any causal SNP as well as number of false-positive SNPs (FP)

### Simulation study 2

Table 3 shows the number of identified SNPs and clumps using logistic regression or r2VIM in the two simulated scenarios. Logistic regression results in the largest total number of SNPs and has only one or two clumps based on false-positive SNPs. In contrast, r2VIM has more false-positive findings, especially when a factor of 1 is used. For the most stringent factor threshold of five, eight and four out of the nine causal SNPs are identified in the two scenarios, however, SNPs in LD with the causal ones are found for all or seven causal SNPs. Surprisingly, for the more difficult scenario with OR of 1.1, LD identifies only five clumps around causal SNPs.

**Table 3:** Number of SNPs and clumps in simulation study 2 with empirical power > 0

						Causal clumps		FP clumps	
OR	*n*	Method	Factor	Total	Causal	no	no SNPs	no	no SNPs
1.3	6000	LR		765	9	9	741	2	24
1.3	6000	r2VIM	1	194	8	9	178	13	16
1.3	6000	r2VIM	3	110	8	9	106	3	4
1.3	6000	r2VIM	5	78	8	9	77	1	1
1.1	20000	LR		106	5	5	105	1	1
1.1	20000	r2VIM	1	104	5	8	62	32	42
1.1	20000	r2VIM	3	43	4	7	30	8	13
1.1	20000	r2VIM	5	26	4	7	21	3	5

Shown are results for logistic regression (LR) and r2VIM. Columns denote odds ratio (OR), sample size (*n*), method, factor for r2VIM, total number of SNPs, number of causal SNPs, number of clumps based on causal SNPs, number of SNPs in clumps based on causal SNPs, number of clumps based on false-positive (FP) SNPs and number of SNPs clumps based on false-positive (FP) SNPs

**Fig. 3:**
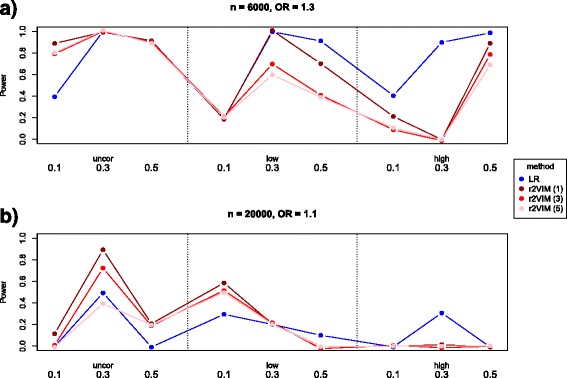
Empirical power of causal SNPs in simulation study 2. Shown are results for logistic regression and r2VIM. Empirical power is shown for the scenario with OR = 1.3 (**a**) and with OR = 1.1 (**b**). Causal SNPs are ordered by LD pattern and MAF. Results for logistic regression are shown in blue, whereas red denotes power of r2VIM (stratified by factor value)

The empirical power for each causal SNP is shown in Fig. 3. Again, power is generally lower for the more difficult simulation scenario with OR of 1.1. Logistic regression usually exhibits larger power for SNPs in low or high LD, but is less powerful for SNPs with low MAF. Interestingly, r2VIM is advantageous for uncorrelated SNPs, especially in the low effect scenario.

In addition, we used the r2VIM results of simulation study 2 to evaluate variability of the relative VIMs and the minimal raw VIM across the ten runs. Results are comparable across the two simulated scenarios. Coefficients of variation (cv) of the relative VIMs strongly depend on the median relative VIM (see Additional file 1: Figure S1). While SNPs which are not selected using a factor threshold of 1 have a median cv of more than 0.5, median cvs drop to about 0.2 for SNPs that are selected by the strict factor threshold of 5. Median cvs of the minimal VIMs are slightly smaller with values of 0.1697 and 0.1899 for the simulations with ORs of 1.3 and 1.1, respectively (see Additional file 1: Table S2).

### Experimental data

**Fig. 4:**
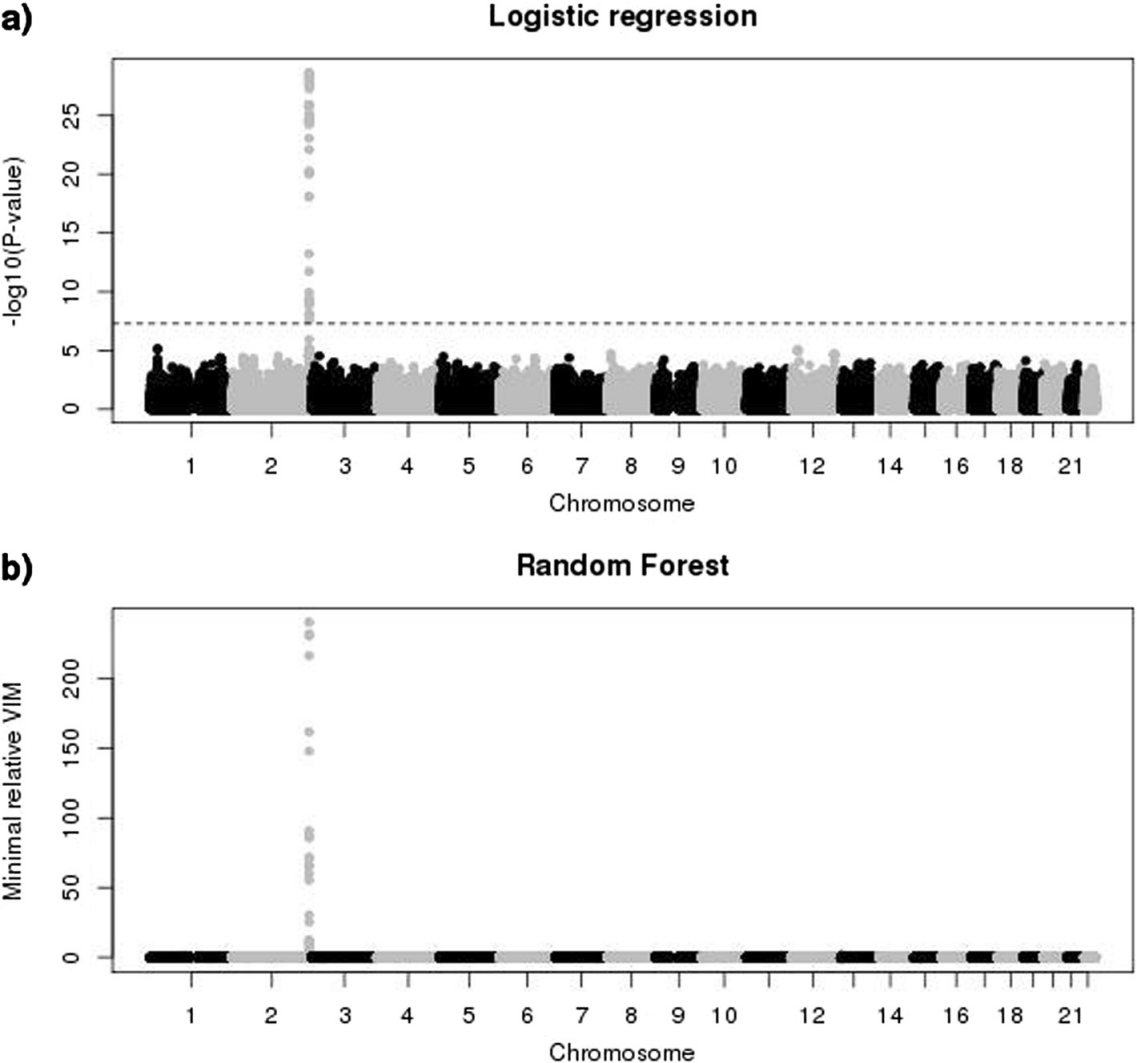
Manhattan plots for TRINITY data set. **a**) *P*-values of logistic regression for each SNP. Dotted line denotes genome-wide significance level of 5*10^−8^. **b**) Minimal relative variable importance (VIM) based on RF analysis for each SNP

The two experimental GWAS data sets have different results. Figure 4 shows a very strong signal on chromosome 2 for the TRINITY study. Ninety-eight SNPs in this region are genome-wide significant with a minimal *p*-value of 2.342*10^−29^ (see Fig. 4a). Forty-two, 35 and 34 SNPs in the same region are selected by r2VIM using factors of 1, 3 and 5, respectively. They have large minimal relative importance scores with a maximum of 240.13 (see Fig. 4b). Additional file 1: Figure S2 compares *P*-values and minimal relative importance scores for SNPs that were selected by either method on chromosome 2. *P*-values are very similar for a long region of 100 kb because of strong linkage disequilibrium, whereas only four SNPs at about 234.33 have very large relative importance scores. Two additional SNPs, one on chromosome 1 and the other one on chromosome 13, are selected with a factor of 1. However, if a more stringent factor is used they are not declared as important and *p*-values of logistic regression are larger than 0.1 for both SNPs. Detailed information about all selected SNPs on chromosome 2 can be found in Additional file 1: Table S3.

**Fig. 5:**
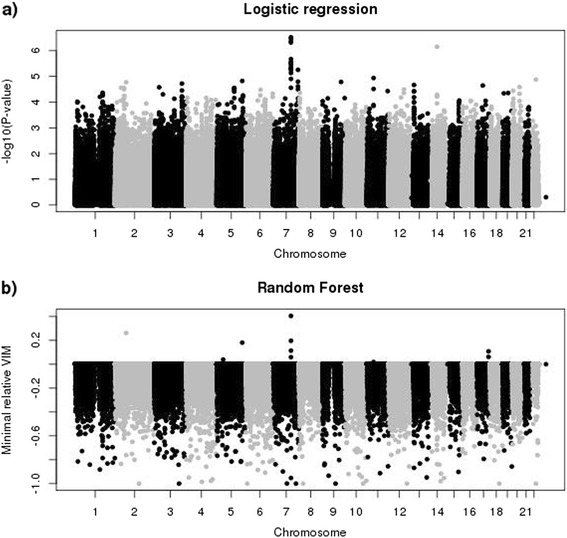
Manhattan plots for AREDS data set. **a**) *P*-values of logistic regression for each SNP. **b**) Minimal relative variable importance (VIM) based on RF analysis for each SNP

Results for the underpowered AREDS study are summarized in Fig. 5. Using logistic regression, no SNP is genome-wide significant and the smallest *p*-value of 3.011*10^−7^ is observed for a SNP on chromosome 7 (see Fig. 5a). Similarly, r2VIM selects none of the SNPs even with the most liberal factor 1 and minimal relative importance scores are much smaller than 1 (see Fig. 5b). Again, SNPs on chromosome 7 have the largest minimal importance scores.

## Discussion

In this work, we presented a new approach for RF to select important variables, i.e. SNPs in GWAS. Evaluations on simulated GWAS data showed that this new method controls the number false-positives and has slightly less power than the standard approach logistic regression.

Further research is needed to evaluate this promising method in more realistic situations. Since this work was designed as a proof-of-concept study we simulated mainly common SNPs with effects that are larger than the ones observed in real studies. A power comparison using more realistic effect sizes, however, would require larger sample sizes so that there is a chance to detect the signal. Another limitation of the current simulation study is the very simple alternative hypothesis with case–control status determined by a small number of SNPs interacting independently. We expect RF in combination with the new variable selection procedure to be more powerful than single SNP analyses in more complex scenarios including gene-gene or gene-environment interactions. Indeed, we showed recently that r2VIM is able to identify interaction effects in situations with purely epistatic effects, i.e. when no marginal effects exist [29].

Our new variable selection method introduces an additional parameter determining the threshold in each run which can be interpreted as follows. A factor of, e.g. 3, means that the relative VIM of a selected variable is three times larger than the relative VIM of a noise variable in each of the independent forests. Our simulations show that a fairly stringent parameter is needed to fully control the number of false-positive SNPs that are identified. However, this approach leads to reduced power. Depending on the costs of follow-up analyses and experiments, more liberal thresholds might be preferred in situations where sensitivity is more important. As an alternative, an optimal threshold for a specific data set could be estimated based on permutations. The threshold corresponding to the desired level of false-positive findings could then be used to analyze the original data set. This would be similar to a family-wise error rate.

In our simulation studies we used a rather broad definition of a true finding and declared only those SNPs as false-positives which were uncorrelated to each of the causal SNPs. This approach is in line with the major goal of a GWAS, namely to find regions in the genome which show association with the phenotype. By design of the arrays used for genotyping, only SNPs in LD with the functionally causal SNP are identified. Further studies including fine-mapping using denser arrays or sequencing are needed to narrow down the association signal.

The focus of this study was on variable selection in a classification setting. However, RF is a very flexible approach than can be used to predict quantitative traits and to estimate probabilities for risk prediction [30]. Modified versions of the permutation importance based on mean square errors are available in current implementations so that our proposed variable selection method can be easily extended to such scenarios.

In addition, these simulations and data analyses all used SNP genotype data as the features (potential predictor variables), which all have the same number of categories [31, 32]. This avoids the known feature selection biases inherent in RF using bootstrap sampling and permutation importance scores when the features vary in their scale of measurement or number of categories. However, in these situations, different implementations of RFs such as cforest [32] or other tree-based regression models such as GUIDE [33] have been shown to correct for these biases. Our approach could be extended to these and other analysis approaches that provide unbiased permutation variable importance metrics.

Variables of different scales are also relevant if known covariates should be included in the analysis. In theory, those covariates could be added to the genotype data and the above mentioned implementions could be used to select the important variables. However, depending on the *mtry* parameter, covariates will have a low probability of being evaluated for a split. An alternative approach would be to use a weighted variable sampling scheme for each split in the tree or always include them in the set of possible split variables.

The principle of our new method to select variables that are important in several runs of the algorithm could, in general, be combined with other machine learning approaches. However, r2VIM is based on several specific features of RF. First, RF calculates importance measures for each single variable and not just for a model based on a specified set of variables such as the overall error rate. Second, the random component in the tree building process leads to slightly different forests if the random number seed is changed. Other suitable machine learning approaches therefore need to provide some measurement of importance or relevance for each variable. To repeatedly perform variable selection, random subsets of the data, e.g. by bootstrapping or subsampling, might be used.

For each hypothesis and each sample size we only simulated ten replicates to reduce computation time. Simulating one replicate and converting the data into appropriate input formats for PLINK and RandomJungle took approximately 4 h on the high performance Biowulf Linux cluster at the National Institutes of Health, Bethesda, MD. We restricted the size of the trees in each forest, so that a single run of RF was performed in about 8 h using two threads. We checked the effect of the depth parameter by generating trees that were only restricted by node size for some of the replicates with similar or slightly worse results (data not shown).

Similarly, we made several decisions regarding the analysis of the two experimental GWAS data sets for illustration purposes. The first was to dichotomize the provided quantitative traits because our simulation study was focused on case–control studies. Although we were still able to identify the strong signal in the TRINITY data, this approach is usually less powerful and therefore not recommended [34]. In the AREDS data set, we reduced the number of SNPs by LD pruning. In a real study we would not recommend to remove SNPs, but rather use RF to select the important variables. In some smaller simulation studies [35, 36], LD seemed to be a problem in identifying the true causal SNP in regions with moderate and high LD, but in our simulations the causal SNP usually had the highest power. However, additional simulation studies are needed to fully explore the effect of LD in a genome-wide setting because our causal SNPs were not located in regions with very high LD and especially not in very long LD blocks.

RF identified a much smaller region in the TRINITY data compared to the large number of SNPs with similar *p*-values based on logistic regression. Three out of the top five SNPs have been reported to be associated with bilirubin levels in several GWAS studies [37–39]. In particular, SNP rs6742078 is in strong LD with the polymorphism UGT1A1*28 in the promoter of the Uridine diphosphate glucuronosyl transferase 1 family, polypeptideA1 gene (*UGT1A1*) [38]. This 2 bp insertion is one of the causal mutations in Gilbert syndrome, a hereditary hyperbilirubinemia [40].

## Conclusions

In conclusion, our new variable selection approach is a promising tool for joint analysis of GWAS data that helps to identify interesting regions for follow up studies while limiting the number of false-positives.

## Additional file

Additional file 1: Figure S1. Supplementary material (figures and tables).
