# Supplementary material for: r2VIM: A new variable selection method for random forests in genome-wide association studies
Source: BioData Min. 2016 Feb 1;9:7. doi: 10.1186/s13040-016-0087-3 (PMC4736152; doi:10.1186/s13040-016-0087-3)
Supplement: Supplementary file 1 — Supplementary material (figures and tables). [file 13040_2016_87_MOESM1_ESM.pdf]

Supplementary material for "r2VIM: A new variable selection method for random forests in genome-wide association studies"

Silke Szymczak Emily R Holzinger Abhijit Dasgupta James D Malley Anne M Molloy James L Mills Lawrence C Brody Dwight Stambolian and Joan E Bailey-Wilson

Contents

1 Supplementary figures **2**

2 Supplementary tables **4**

1 Supplementary figures

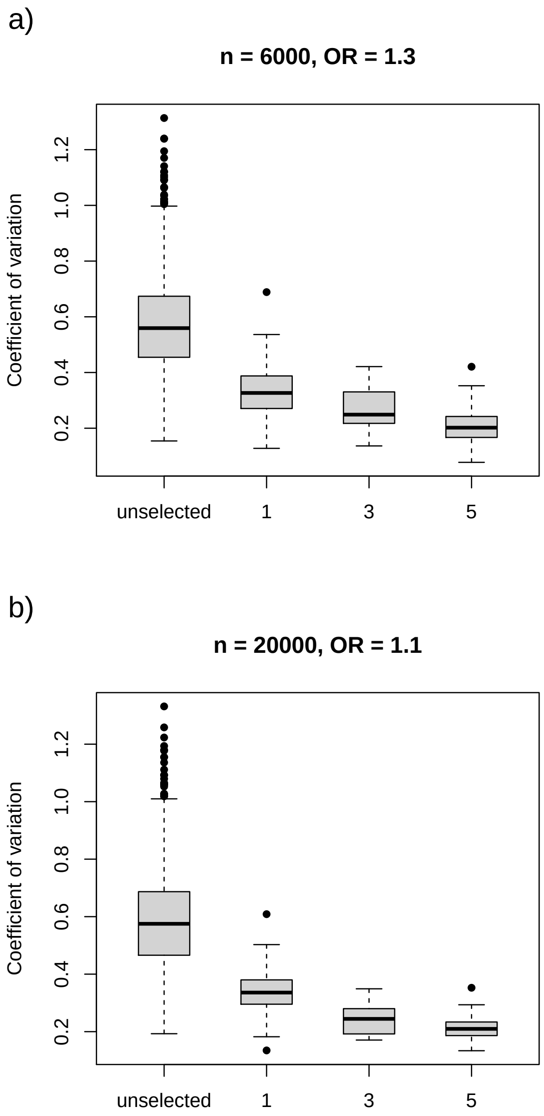

Supplementary Figure 1: Coefficient of variation (cv) of relative variable importance (VIM). Boxplots summarize distributions of cv for each SNP with a minimal relative VIM ¿ 0 (unselected), 1, 3 or 5 across all ten replicates of simulation data for the scenario with a) OR = 1.3 and total sample size = 6,000 and b) OR = 1.1 and total sample size = 20,000.

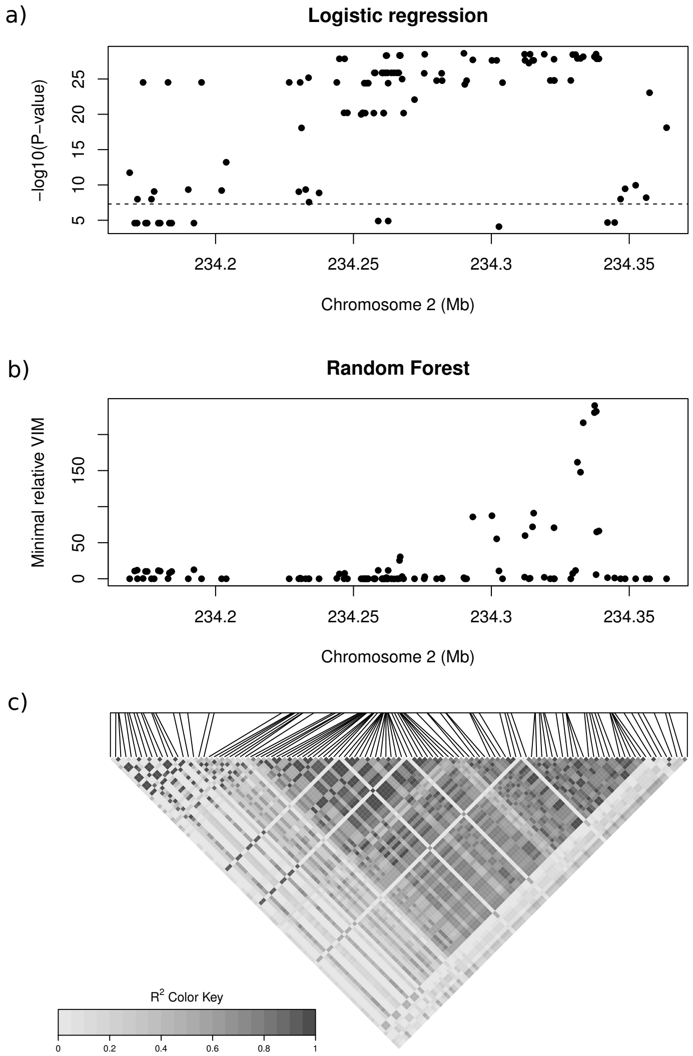

Supplementary Figure 2: Regional plot of region on chromosome 2 for TRINITY data set. a) P-values of logistic regression for each SNP. Dotted line denotes genome-wide significance level of $5 * 10^{-8}$. b) Minimal relative variable importance (VIM) for each SNP. c) Pairwise linkage disequilibirum (r^2) for all SNPs in region.

2 Supplementary tables

Supplementary Table 1: Detailed results for all SNPs selected by at least one method under the simulated alternative hypothesis of simulation study 1. Table shows SNP identifier in chromosome and position notation, characterization of SNP (clumped = in LD with causal SNP on the same chromosome, fp = false positive), pairwise LD (r^2) for clumped SNPs to causal SNP on the same chromosome, empirical power for logistic regression (LR) and random forest (RF) in the different scenarios (different sample sizes (n) as well as $mtry$ parameters and factors (f) for RF).

SNP	info	LD	LR $n=2000$	LR $n=6000$	RF $n=2000$ $mtry=20\%$ $f=1$	RF $n=2000$ $mtry=20\%$ $f=3$	RF $n=2000$ $mtry=20\%$ $f=5$	RF $n=2000$ $mtry=50\%$ $f=1$	RF $n=2000$ $mtry=50\%$ $f=3$	RF $n=2000$ $mtry=50\%$ $f=5$	RF $n=6000$ $mtry=20\%$ $f=1$	RF $n=6000$ $mtry=20\%$ $f=3$	RF $n=6000$ $mtry=20\%$ $f=5$	RF $n=6000$ $mtry=50\%$ $f=1$	RF $n=6000$ $mtry=50\%$ $f=3$	RF $n=6000$ $mtry=50\%$ $f=5$
1-240798089	clumped	0.113	0.0	0.1	0.0	0.0	0.0	0.0	0.0	0.0	0.0	0.0	0.0	0.0	0.0	0.0
1-240799543	causal		0.6	1.0	0.6	0.5	0.5	0.6	0.5	0.5	1.0	1.0	1.0	1.0	1.0	1.0
2-49887106	fp		0.0	0.0	0.0	0.0	0.0	0.0	0.0	0.0	0.0	0.0	0.0	0.1	0.0	0.0
2-49898423	fp		0.0	0.0	0.0	0.0	0.0	0.0	0.0	0.0	0.1	0.0	0.0	0.1	0.1	0.0
3-2770509	causal		0.1	1.0	0.6	0.3	0.1	0.6	0.4	0.2	0.9	0.9	0.8	0.9	0.9	0.8
3-34846048	fp		0.0	0.0	0.0	0.0	0.0	0.1	0.0	0.0	0.0	0.0	0.0	0.0	0.0	0.0
3-68296488	fp		0.0	0.0	0.0	0.0	0.0	0.0	0.0	0.0	0.0	0.0	0.0	0.1	0.0	0.0
3-168581491	fp		0.0	0.0	0.0	0.0	0.0	0.0	0.0	0.0	0.0	0.0	0.0	0.1	0.0	0.0
3-175118665	fp		0.0	0.0	0.0	0.0	0.0	0.0	0.0	0.0	0.0	0.0	0.0	0.1	0.0	0.0
4-5078789	fp		0.0	0.0	0.0	0.0	0.0	0.1	0.0	0.0	0.0	0.0	0.0	0.0	0.0	0.0
4-172050379	fp		0.0	0.0	0.0	0.0	0.0	0.1	0.0	0.0	0.0	0.0	0.0	0.0	0.0	0.0
5-116549323	fp		0.0	0.0	0.0	0.0	0.0	0.0	0.0	0.0	0.0	0.0	0.0	0.1	0.0	0.0
5-129723516	clumped	0.325	0.0	0.3	0.0	0.0	0.0	0.0	0.0	0.0	0.0	0.0	0.0	0.0	0.0	0.0
5-129759110	clumped	0.664	0.2	1.0	0.1	0.1	0.1	0.1	0.1	0.1	0.5	0.2	0.2	0.3	0.1	0.1
5-129797129	clumped	0.657	0.3	1.0	0.1	0.1	0.0	0.1	0.0	0.0	0.5	0.3	0.2	0.2	0.1	0.0
5-129820038	clumped	0.183	0.0	0.4	0.0	0.0	0.0	0.0	0.0	0.0	0.0	0.0	0.0	0.0	0.0	0.0
5-129846173	clumped	0.299	0.0	0.3	0.0	0.0	0.0	0.0	0.0	0.0	0.0	0.0	0.0	0.0	0.0	0.0
5-129875072	clumped	0.483	0.1	1.0	0.0	0.0	0.0	0.0	0.0	0.0	0.7	0.3	0.3	0.3	0.3	0.1
5-129888054	clumped	0.141	0.0	0.1	0.0	0.0	0.0	0.0	0.0	0.0	0.0	0.0	0.0	0.0	0.0	0.0
5-129891761	clumped	0.300	0.0	0.1	0.0	0.0	0.0	0.0	0.0	0.0	0.0	0.0	0.0	0.0	0.0	0.0
5-129921388	clumped	0.148	0.0	0.1	0.0	0.0	0.0	0.0	0.0	0.0	0.0	0.0	0.0	0.0	0.0	0.0
5-129925322	clumped	0.354	0.0	0.8	0.0	0.0	0.0	0.0	0.0	0.0	0.1	0.0	0.0	0.1	0.0	0.0
5-129949992	clumped	0.807	0.5	1.0	0.3	0.3	0.2	0.3	0.3	0.3	0.9	0.7	0.5	0.8	0.5	0.4
5-130017287	clumped	0.548	0.1	0.9	0.0	0.0	0.0	0.0	0.0	0.0	0.0	0.0	0.0	0.1	0.0	0.0
5-130022414	clumped	0.928	0.5	1.0	0.3	0.3	0.2	0.4	0.2	0.1	1.0	1.0	1.0	1.0	0.9	0.9
5-130032562	clumped	0.551	0.1	0.9	0.1	0.0	0.0	0.0	0.0	0.0	0.0	0.0	0.0	0.0	0.0	0.0
5-130034638	clumped	0.928	0.5	1.0	0.4	0.3	0.1	0.3	0.3	0.2	1.0	1.0	1.0	1.0	0.9	0.9
5-130084469	clumped	0.548	0.1	0.9	0.0	0.0	0.0	0.0	0.0	0.0	0.0	0.0	0.0	0.1	0.0	0.0
5-130088218	clumped	0.922	0.5	1.0	0.4	0.3	0.1	0.3	0.1	0.1	1.0	1.0	1.0	1.0	0.9	0.7
5-130096260	clumped	0.757	0.1	1.0	0.1	0.1	0.1	0.1	0.1	0.1	0.6	0.3	0.3	0.4	0.3	0.3
5-130102023	clumped	0.987	0.6	1.0	0.5	0.4	0.4	0.5	0.4	0.2	1.0	1.0	1.0	1.0	1.0	1.0
5-130104076	causal		0.7	1.0	0.5	0.4	0.3	0.6	0.5	0.5	1.0	1.0	1.0	1.0	1.0	1.0
5-130111154	clumped	0.918	0.5	1.0	0.3	0.3	0.2	0.3	0.2	0.1	1.0	1.0	1.0	0.9	0.6	0.6

Supplementary Table 1: Continued

5-130113901	clumped	0.904	0.5	1.0	0.4	0.1	0.1	0.1	0.1	0.1	1.0	1.0	0.9	0.7	0.3	0.3
5-130130195	clumped	0.879	0.5	1.0	0.3	0.2	0.1	0.2	0.2	0.1	0.9	0.9	0.7	0.6	0.6	0.3
5-130136097	clumped	0.885	0.5	1.0	0.2	0.2	0.1	0.2	0.1	0.1	1.0	0.9	0.9	0.8	0.5	0.5
5-130140680	clumped	0.570	0.0	0.9	0.0	0.0	0.0	0.0	0.0	0.0	0.0	0.0	0.0	0.0	0.0	0.0
5-130147567	clumped	0.849	0.4	1.0	0.2	0.1	0.1	0.1	0.0	0.0	0.9	0.9	0.5	0.5	0.3	0.2
5-130148617	clumped	0.856	0.4	1.0	0.3	0.0	0.0	0.2	0.0	0.0	0.9	0.8	0.5	0.4	0.4	0.3
5-130149253	clumped	0.856	0.4	1.0	0.2	0.1	0.0	0.1	0.0	0.0	0.8	0.6	0.4	0.4	0.4	0.4
5-130187390	clumped	0.432	0.0	0.7	0.0	0.0	0.0	0.0	0.0	0.0	0.0	0.0	0.0	0.0	0.0	0.0
5-130195616	clumped	0.523	0.2	1.0	0.0	0.0	0.0	0.0	0.0	0.0	0.0	0.0	0.0	0.1	0.0	0.0
5-130195731	clumped	0.290	0.0	0.2	0.0	0.0	0.0	0.0	0.0	0.0	0.0	0.0	0.0	0.0	0.0	0.0
5-130195812	clumped	0.515	0.2	1.0	0.1	0.0	0.0	0.1	0.0	0.0	0.2	0.0	0.0	0.1	0.1	0.0
5-130219200	clumped	0.312	0.0	0.4	0.0	0.0	0.0	0.0	0.0	0.0	0.0	0.0	0.0	0.0	0.0	0.0
5-130242429	clumped	0.390	0.1	0.7	0.0	0.0	0.0	0.0	0.0	0.0	0.0	0.0	0.0	0.0	0.0	0.0
5-130272586	clumped	0.281	0.0	0.5	0.0	0.0	0.0	0.0	0.0	0.0	0.0	0.0	0.0	0.0	0.0	0.0
5-130344771	clumped	0.321	0.0	0.4	0.0	0.0	0.0	0.0	0.0	0.0	0.0	0.0	0.0	0.0	0.0	0.0
5-130359215	clumped	0.315	0.0	0.3	0.0	0.0	0.0	0.0	0.0	0.0	0.0	0.0	0.0	0.0	0.0	0.0
5-130373830	clumped	0.315	0.0	0.3	0.0	0.0	0.0	0.0	0.0	0.0	0.0	0.0	0.0	0.0	0.0	0.0
5-130446419	clumped	0.110	0.0	0.2	0.0	0.0	0.0	0.0	0.0	0.0	0.0	0.0	0.0	0.0	0.0	0.0
5-130497428	clumped	0.143	0.0	0.3	0.0	0.0	0.0	0.0	0.0	0.0	0.0	0.0	0.0	0.0	0.0	0.0
7-45955029	clumped	0.267	0.0	1.0	0.0	0.0	0.0	0.0	0.0	0.0	0.4	0.2	0.0	0.2	0.0	0.0
7-45965795	clumped	0.112	0.0	0.1	0.0	0.0	0.0	0.0	0.0	0.0	0.0	0.0	0.0	0.0	0.0	0.0
7-45977173	clumped	0.125	0.0	0.3	0.0	0.0	0.0	0.0	0.0	0.0	0.0	0.0	0.0	0.0	0.0	0.0
7-45979023	clumped	0.487	0.4	1.0	0.3	0.2	0.2	0.4	0.2	0.2	1.0	0.9	0.9	0.8	0.8	0.7
7-45980751	clumped	0.506	0.4	1.0	0.3	0.2	0.2	0.2	0.2	0.2	1.0	1.0	0.8	0.9	0.8	0.8
7-45981123	clumped	0.112	0.0	0.2	0.0	0.0	0.0	0.0	0.0	0.0	0.0	0.0	0.0	0.0	0.0	0.0
7-45984645	clumped	0.218	0.0	0.8	0.0	0.0	0.0	0.0	0.0	0.0	0.3	0.1	0.0	0.2	0.1	0.1
7-45984820	causal		0.7	1.0	0.8	0.6	0.6	0.7	0.6	0.6	1.0	1.0	1.0	1.0	1.0	1.0
7-45985290	clumped	0.218	0.0	0.8	0.0	0.0	0.0	0.0	0.0	0.0	0.3	0.2	0.0	0.2	0.1	0.1
7-46006544	clumped	0.117	0.0	0.3	0.0	0.0	0.0	0.0	0.0	0.0	0.1	0.0	0.0	0.0	0.0	0.0
7-46016304	clumped	0.124	0.0	0.2	0.0	0.0	0.0	0.0	0.0	0.0	0.0	0.0	0.0	0.0	0.0	0.0
7-46018146	clumped	0.186	0.0	0.1	0.0	0.0	0.0	0.0	0.0	0.0	0.0	0.0	0.0	0.0	0.0	0.0
7-46021684	clumped	0.245	0.1	1.0	0.1	0.0	0.0	0.0	0.0	0.0	0.4	0.3	0.1	0.2	0.1	0.0
7-46030400	clumped	0.079	0.0	0.1	0.0	0.0	0.0	0.0	0.0	0.0	0.0	0.0	0.0	0.0	0.0	0.0
7-46042692	clumped	0.079	0.0	0.1	0.0	0.0	0.0	0.0	0.0	0.0	0.0	0.0	0.0	0.0	0.0	0.0
7-135840925	fp		0.0	0.0	0.0	0.0	0.0	0.1	0.0	0.0	0.0	0.0	0.0	0.0	0.0	0.0
8-63072917	fp		0.0	0.0	0.1	0.0	0.0	0.1	0.0	0.0	0.0	0.0	0.0	0.0	0.0	0.0
8-63083535	fp		0.0	0.0	0.1	0.0	0.0	0.0	0.0	0.0	0.0	0.0	0.0	0.0	0.0	0.0
8-138887431	fp		0.0	0.0	0.0	0.0	0.0	0.0	0.0	0.0	0.0	0.0	0.0	0.1	0.0	0.0
10-17040116	fp		0.0	0.0	0.0	0.0	0.0	0.1	0.0	0.0	0.0	0.0	0.0	0.0	0.0	0.0
10-116463434	fp		0.0	0.0	0.0	0.0	0.0	0.0	0.0	0.0	0.0	0.0	0.0	0.1	0.0	0.0
11-103924576	clumped	0.044	0.0	0.1	0.0	0.0	0.0	0.0	0.0	0.0	0.0	0.0	0.0	0.0	0.0	0.0
11-103936469	clumped	0.044	0.0	0.1	0.0	0.0	0.0	0.0	0.0	0.0	0.0	0.0	0.0	0.0	0.0	0.0
11-103956277	clumped	0.044	0.0	0.1	0.0	0.0	0.0	0.0	0.0	0.0	0.0	0.0	0.0	0.0	0.0	0.0
11-103957628	clumped	0.044	0.0	0.1	0.0	0.0	0.0	0.0	0.0	0.0	0.0	0.0	0.0	0.0	0.0	0.0
11-103959987	causal		0.0	1.0	0.0	0.0	0.0	0.0	0.0	0.0	1.0	0.9	0.7	0.9	0.9	0.6
11-103964427	clumped	0.253	0.0	0.1	0.0	0.0	0.0	0.0	0.0	0.0	0.0	0.0	0.0	0.0	0.0	0.0
11-103965133	clumped	0.478	0.0	0.4	0.0	0.0	0.0	0.0	0.0	0.0	0.0	0.0	0.0	0.1	0.0	0.0
12-5870249	fp		0.0	0.0	0.0	0.0	0.0	0.0	0.0	0.0	0.0	0.0	0.0	0.1	0.0	0.0
13-106651770	fp		0.0	0.0	0.0	0.0	0.0	0.0	0.0	0.0	0.0	0.0	0.0	0.1	0.0	0.0
14-22977669	fp		0.0	0.0	0.0	0.0	0.0	0.0	0.0	0.0	0.1	0.0	0.0	0.1	0.0	0.0
14-67310195	clumped	0.128	0.0	0.1	0.0	0.0	0.0	0.0	0.0	0.0	0.0	0.0	0.0	0.0	0.0	0.0
14-67427290	clumped	0.128	0.1	0.7	0.2	0.1	0.0	0.3	0.1	0.0	0.4	0.2	0.2	0.3	0.3	0.1
14-67453858	clumped	0.375	0.4	1.0	0.5	0.4	0.1	0.5	0.3	0.2	0.8	0.6	0.3	0.6	0.3	0.3
14-67463012	causal		0.3	1.0	0.5	0.4	0.1	0.4	0.4	0.2	1.0	0.9	0.9	1.0	0.8	0.7

Supplementary Table 1: Continued

14-67527235	clumped	0.510	0.3	1.0	0.5	0.3	0.2	0.4	0.4	0.3	0.9	0.8	0.4	0.8	0.6	0.4
14-67536019	clumped	0.510	0.3	1.0	0.6	0.4	0.3	0.4	0.3	0.2	1.0	0.8	0.5	0.9	0.6	0.4
14-67606802	clumped	0.517	0.0	0.7	0.1	0.0	0.0	0.1	0.0	0.0	0.2	0.0	0.0	0.1	0.0	0.0
14-67608948	clumped	0.129	0.0	0.2	0.0	0.0	0.0	0.0	0.0	0.0	0.0	0.0	0.0	0.0	0.0	0.0
14-67626594	clumped	0.517	0.0	0.7	0.1	0.0	0.0	0.1	0.0	0.0	0.3	0.0	0.0	0.0	0.0	0.0
14-67760538	clumped	0.149	0.0	0.5	0.0	0.0	0.0	0.0	0.0	0.0	0.3	0.2	0.0	0.3	0.2	0.0
15-69905995	fp		0.0	0.0	0.1	0.0	0.0	0.1	0.0	0.0	0.0	0.0	0.0	0.0	0.0	0.0
17-9783855	clumped	0.203	0.0	0.1	0.0	0.0	0.0	0.0	0.0	0.0	0.0	0.0	0.0	0.0	0.0	0.0
17-9807099	causal		0.1	1.0	0.0	0.0	0.0	0.1	0.0	0.0	0.8	0.5	0.5	0.8	0.6	0.5
18-34464502	clumped	0.025	0.0	0.1	0.0	0.0	0.0	0.0	0.0	0.0	0.0	0.0	0.0	0.0	0.0	0.0
18-34515400	clumped	0.025	0.0	0.1	0.0	0.0	0.0	0.0	0.0	0.0	0.0	0.0	0.0	0.0	0.0	0.0
18-34532038	clumped	0.025	0.0	0.1	0.0	0.0	0.0	0.0	0.0	0.0	0.0	0.0	0.0	0.0	0.0	0.0
18-34557276	clumped	0.025	0.0	0.1	0.0	0.0	0.0	0.0	0.0	0.0	0.0	0.0	0.0	0.0	0.0	0.0
18-34635606	clumped	0.003	0.0	0.1	0.0	0.0	0.0	0.0	0.0	0.0	0.0	0.0	0.0	0.0	0.0	0.0
18-34645639	causal		0.2	1.0	0.6	0.4	0.2	0.7	0.4	0.4	1.0	0.9	0.8	1.0	1.0	0.9
18-34722503	clumped	0.025	0.0	0.1	0.0	0.0	0.0	0.0	0.0	0.0	0.0	0.0	0.0	0.0	0.0	0.0
18-34723643	clumped	0.026	0.0	0.1	0.0	0.0	0.0	0.0	0.0	0.0	0.0	0.0	0.0	0.0	0.0	0.0
18-34731805	clumped	0.004	0.0	0.1	0.0	0.0	0.0	0.0	0.0	0.0	0.0	0.0	0.0	0.0	0.0	0.0
18-59729644	fp		0.0	0.0	0.1	0.0	0.0	0.1	0.1	0.0	0.0	0.0	0.0	0.0	0.0	0.0
22-28301743	clumped	0.506	0.0	0.4	0.0	0.0	0.0	0.0	0.0	0.0	0.2	0.0	0.0	0.1	0.0	0.0
22-28317401	clumped	0.155	0.0	0.1	0.0	0.0	0.0	0.0	0.0	0.0	0.0	0.0	0.0	0.0	0.0	0.0
22-28337711	clumped	0.837	0.1	0.9	0.0	0.0	0.0	0.0	0.0	0.0	0.4	0.4	0.2	0.5	0.2	0.2
22-28372703	clumped	0.337	0.0	0.2	0.0	0.0	0.0	0.0	0.0	0.0	0.0	0.0	0.0	0.0	0.0	0.0
22-28380588	clumped	0.848	0.0	0.9	0.0	0.0	0.0	0.0	0.0	0.0	0.5	0.3	0.3	0.5	0.4	0.3
22-28393540	clumped	0.864	0.1	1.0	0.0	0.0	0.0	0.0	0.0	0.0	0.5	0.3	0.3	0.5	0.4	0.1
22-28411918	clumped	0.970	0.1	1.0	0.1	0.1	0.0	0.0	0.0	0.0	0.8	0.6	0.4	0.8	0.5	0.4
22-28444744	clumped	0.448	0.0	0.5	0.0	0.0	0.0	0.0	0.0	0.0	0.1	0.1	0.1	0.2	0.1	0.1
22-28449353	clumped	0.173	0.0	0.1	0.0	0.0	0.0	0.0	0.0	0.0	0.0	0.0	0.0	0.0	0.0	0.0
22-28454655	clumped	0.438	0.0	0.6	0.0	0.0	0.0	0.0	0.0	0.0	0.0	0.0	0.0	0.0	0.0	0.0
22-28469630	causal		0.1	1.0	0.1	0.1	0.1	0.1	0.1	0.1	0.8	0.6	0.5	0.7	0.6	0.5
22-28550006	clumped	0.228	0.0	0.1	0.0	0.0	0.0	0.0	0.0	0.0	0.1	0.0	0.0	0.1	0.0	0.0
22-34133758	fp		0.0	0.0	0.0	0.0	0.0	0.0	0.0	0.0	0.1	0.0	0.0	0.1	0.0	0.0
22-44466550	fp		0.0	0.0	0.0	0.0	0.0	0.0	0.0	0.0	0.0	0.0	0.0	0.1	0.0	0.0

Supplementary Table 2: Information about minimal importance scores in simulation study 2. Table shows mean, standard deviation (sd) and coefficient of variation (cv) of the minimal importance score across the ten RF runs for each replicate (rep) in both simulated scenarios described by odds ratio (OR) and total sample size (n).

OR	n	rep	mean	sd	cv
1.3	6000	1	-3.51e-05	1.18e-05	0.335
1.3	6000	2	-3.33e-05	8.63e-06	0.259
1.3	6000	3	-3.15e-05	7.46e-06	0.237
1.3	6000	4	-3.24e-05	4.64e-06	0.143
1.3	6000	5	-3.41e-05	9.14e-06	0.268
1.3	6000	6	-3.14e-05	4.03e-06	0.128
1.3	6000	7	-2.91e-05	5.72e-06	0.196
1.3	6000	8	-2.85e-05	2.01e-06	0.071
1.3	6000	9	-3.10e-05	3.20e-06	0.103
1.3	6000	10	-3.61e-05	4.90e-06	0.136
1.1	20000	1	-2.34e-05	5.55e-06	0.237
1.1	20000	2	-2.00e-05	3.50e-06	0.175
1.1	20000	3	-2.42e-05	5.38e-06	0.223
1.1	20000	4	-2.29e-05	5.11e-06	0.224
1.1	20000	5	-1.96e-05	3.78e-06	0.193
1.1	20000	6	-2.08e-05	4.16e-06	0.200
1.1	20000	7	-1.85e-05	2.52e-06	0.136
1.1	20000	8	-2.32e-05	4.14e-06	0.178
1.1	20000	9	-2.17e-05	3.80e-06	0.175
1.1	20000	10	-2.15e-05	4.01e-06	0.187

Supplementary Table 3: Information about SNPs on chromosome 2 selected by the new variable selection method in the TRINITY GWAS. Table gives SNP identifier, chromosome, position in base pairs, p-value of logistic regression and minimal relative variable importance.

SNP	chr	position	p-value	minimal relative importance
rs17868298	2	234170654	2.56e-05	10.71
rs17868299	2	234171547	2.56e-05	11.86
rs17864666	2	234174596	2.56e-05	10.17
rs7563478	2	234175185	2.56e-05	10.11
rs17862835	2	234179252	2.56e-05	11.30
rs1551286	2	234179894	2.56e-05	10.55
rs17864669	2	234183253	2.56e-05	8.01
rs17868306	2	234184076	2.56e-05	10.03
rs17864670	2	234192137	2.56e-05	12.54
rs2741045	2	234244879	1.39e-28	6.73
rs2602376	2	234246790	1.39e-28	7.49
rs17864689	2	234258973	1.29e-05	11.62
rs10168416	2	234261826	4.88e-29	1.41
rs10173355	2	234262060	4.88e-29	1.56
rs17862859	2	234262581	1.29e-05	11.56
rs1105880	2	234266704	4.63e-29	25.38
rs1105879	2	234266941	4.63e-29	30.39
rs7592624	2	234267645	1.04e-25	3.04
rs17863787	2	234275833	3.22e-29	2.82
rs6744284	2	234290036	2.34e-29	1.43
rs871514	2	234293268	1.96e-28	85.76
rs4294999	2	234300206	2.33e-28	87.41
rs2008595	2	234301931	2.33e-28	55.35
rs17868336	2	234302745	8.10e-05	10.89
rs6722076	2	234312056	3.15e-29	2.25
rs6431628	2	234312217	2.33e-28	59.80
rs4663963	2	234314932	2.33e-28	71.99
rs4663965	2	234315343	2.33e-28	90.96
rs11695484	2	234319188	3.15e-29	2.03
rs10179091	2	234322722	1.61e-28	70.79
rs6714634	2	234329504	3.55e-29	7.32
rs10929302	2	234330521	3.55e-29	11.43
rs4399719	2	234331200	1.23e-28	161.56
rs3755319	2	234332321	1.23e-28	147.74
rs887829	2	234333309	7.08e-29	216.32
rs6742078	2	234337378	7.08e-29	230.41
rs4148324	2	234337461	7.08e-29	240.13
rs3771341	2	234337978	3.01e-29	5.72
rs4148325	2	234338048	7.08e-29	232.00
rs4148326	2	234338201	1.38e-28	64.90
rs4663971	2	234338991	1.38e-28	66.25
rs6431630	2	234342125	2.12e-05	1.49
rs11888492	2	234344713	2.12e-05	1.08
